# Turbidity and RI Dependency of a Polymer Optical Fiber-Based Chromatic Sensor

**DOI:** 10.3390/s20010019

**Published:** 2019-12-19

**Authors:** Daniel P. Duarte, Rogério N. Nogueira, Lucia Bilro

**Affiliations:** 1 Instituto de Telecomunicações, 3810-193 Aveiro, Portugal; rnogueira@av.it.pt; 2 Physics Department, Aveiro University, 3810-193 Aveiro, Portugal; lucia.bilro@av.it.pt

**Keywords:** chromatic sensor, refractive index, turbidity, in-line, colorimeter, optical fiber

## Abstract

An in-line and real time chromatic sensor for liquids based on plastic optical fiber was developed. It uses an air gap, fiber to fiber, transmission principle. Its dependency to turbidity and refractive index is studied and characterized. This information will provide the necessary knowledge for future implementation of more complex auto-compensations routines. Due to the predictable behavior of the sensor to variations of turbidity and refractive index, it is shown that a posterior compensation could be applied for the discrimination of color. The real-time color sensor can be used in different turbid liquids and contain different refractive indices.

## 1. Introduction

In-line and online sensors for liquids are gradually gaining an increased market share with the advent of the internet of things in an ever-interconnected world. For example, in an industrial environment, they allow a timely reaction to any production flaws that can occur. Storing the historical data obtained, in the perspective of a Big Data analysis, can also help to identify systematic or regular flaws that could not be perceived easily by standard control engineering [1]. In the same way, quality control in liquids is essential in many sectors, from industry to biology. One of these quality control parameters that can be monitored in liquids is the color [2,3,4].

Measuring color for water based non-turbid solutions is a simple process and well documented in literature with several commercial solutions available, like spectrophotometers and colorimeters [5,6,7]. State of the art developments show there are some approaches for the assessment of liquid color that tries to create low-cost, portable and easy to use devices. This is the case of a very simple sample holder-based colorimeter, constructed using LEGO bricks that has a single wavelength light-emitting diode (LED) as light source [8] or a more complex colorimeter for chemistry laboratories that uses three LEDs of 640, 524 and 470 nm wavelengths presenting the transmittance values to an integrated liquid crystal display (LCD) [9]. This was also the approach taken by Jiménez-Márquez et al. for a solution to be used for the monitoring of the maceration of red wine [10]. The transmitted light of the three red-green-blue (RGB) LEDs of the wavelength of interest is distributed throughout the measurement cell and the measurement of the transmittance values are independently determined by a row of three photodiodes distributed in the same way as in the LEDs. Another approach presented by Zhou et al. [11] uses filters in the light path between the white LEDs sources and the sample holder instead of different color LEDs. The only in situ and online measurement device found in literature ready for color measurement was presented by Novo et al. [12,13] and Shrake et al. [14] for wine monitoring. Novo uses the advantages of polymer optical fiber (POF) miniaturization and maneuverability to create four separate air-gap regions between two fiber extremities in a sensor head structure where the wine to be measured will flow. One of the fibers will be connected to an LED and the other to a photodetector to detect the transmittance. Shrake also uses POF for light guiding of 420, 470, 503, 525, 565 and 630 nm LEDs but this light is directly captured by photodiodes instead of a fiber.

Although there are developed solutions for color monitoring like those presented here, these devices have a major handicap of needing cleared liquids for color determination. When turbidity and refractive index variations are present or occur, a dependency on these parameters with changes in the measurement signal will be observed. This means that one cannot measure color without considering the effects of refractive index changes and the turbidity in the liquid.

In this work, a study of an optical color sensor for liquids, based on the transmission of light from fiber-to-fiber, is presented. Measurement of color is performed due to the absorbance of light from the liquid. Its characterization to turbidity and refractive index dependency is also undertaken. Possible compensation for turbidity and refractive index present in the solution is discussed for future developments.

## 2. Sensor Design

**Figure 1:**
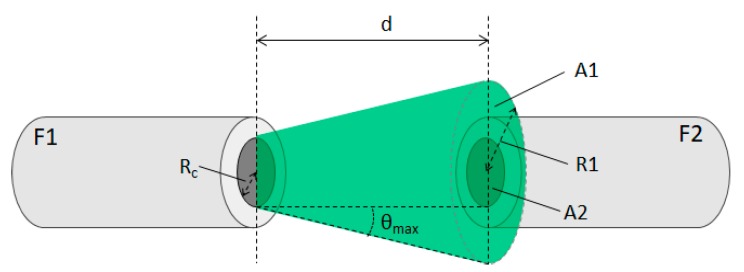
Air-gap transmission of light between two perfectly aligned fibers.

The chromatic sensor is based on the air-gap, multimode fiber-to-fiber, transmission principle. A schematic view of this principle is presented in Figure 1 where a geometric model for a multimode fiber can be used [15].

The light emitted by one of the fibers, identified as *F1*, will propagate in a conical shape until it reaches the second fiber, *F2*, at a distance *d*. Because of its increasing conical shape, only a fraction of light reaches *F2*. The ratio of the received light power *P2*, by the emitted power, *P1*, will be equal to the ratio of the receiving area *A2*, with the light emitting area *A1* when it reaches the fiber *F2*. Considering that the light energy is radially uniform through the fiber, the fraction of light power that reaches *F2* will be mostly dependent on geometric factors like the distance between fibers (*d*) and the fiber core radius (*R_c_*) [15]:

(1) $$P_{2}=P_{1}\frac{A_{2}}{A_{1}}=P_{1}\frac{R_{c}}{R_{c}+d\times \tan\left(\theta_{max}\right)}$$

*θ_max_* is the aperture angle that will depend on the refractive index of the fiber core (*n_co_*) and cladding (*n_cl_*), but also it will vary with refractive index of the external medium (*n_ext_*) as,

(2) $$\theta_{max}=\arcsin\left(\frac{\sqrt{n_{co}{}^{2}-n_{cl}{}^{2}}}{n_{ext}}\right)$$

When submerged in solutions where there is the existence of colored or suspended particles, phenomena of scattering or absorption of light will occur. This is described by the Beer-Lambert law that states that the attenuation (*A*) of light have a logarithmic relation with the transmission light intensity, in the presence of the attenuation agent (*I*) and without the presence of it (*I_0_*):

(3) $$A=-log_{10}\left(\frac{I}{I_{0}}\right)$$

**Figure 2:**
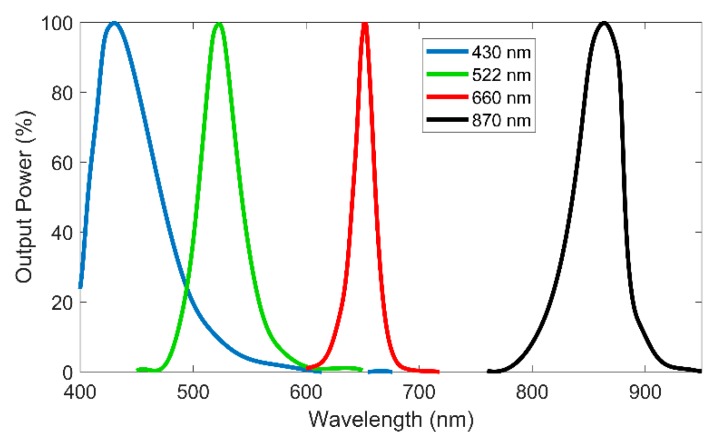
Output power spectrum of the light-emitting diodes (LEDs) used in the sensor identified by its central wavelength in the legend.

For color measurement, the sensor uses 3 LEDs from Industrial Fiber Optics with central wavelengths of 430 nm (IF E92A), 522 nm (IF E93), and 660 nm (IF E97). These wavelengths were chosen to give a wide color perception of the solutions to measure, being the same or near to the usually used in colorimeters found in the market [16]. A fourth LED with wavelength of 870 nm (IF E91D) in the infrared region and unaffected by colored solutions is also used to measure turbidity. The light spectrum of each LED is presented in Figure 2.

**Figure 3:**
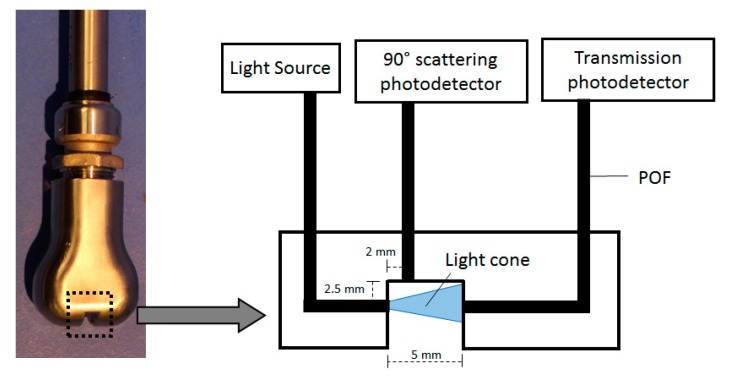
Sensing head photograph and schematic of its operation principle.

The 870 nm LED will be a key element for turbidity compensation. All the LEDs are controlled by an electronic board connected to a 4 × 4 POF coupler with splitting ratio of 25:25:25:25%, all in the same side. One of the outputs of the coupler is used as the source light to a stainless-steel sensing head, where the solution to measure will flow. The board has three photodetectors, one for the transmission measurement, a second directly connected to a second output of the coupler for optical drifts numeric compensation, and the third will be associated to a POF for scattering measurements also used for turbidity measurements (Figure 3). When the measuring mode starts, the board will sequentially turn on and off each light source for a second and register the data obtained from the photodetectors. A full measurement will have information from the three photodetectors and from the four-light source, counting a total of 12 values. This information is then sent to an online server by a wireless module for measurement calculation using the calibrations. The POF used was the ESKA^TM^ series model GHCP4001 from Mitsubishi Rayon Co., Ltd. (Tokyo, Japan).

## 3. Color and Turbidity

**Figure 4:**
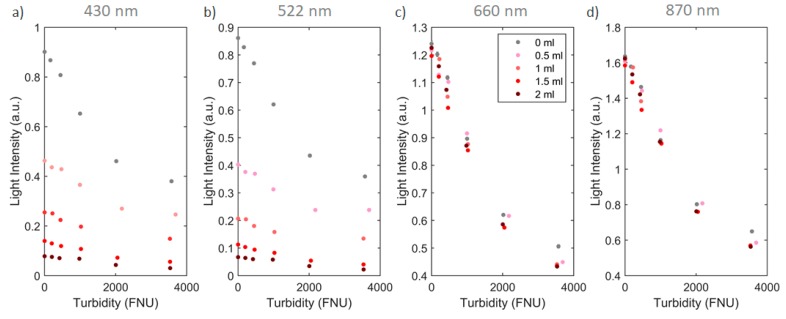
Light intensity variation with the increase of turbidity (0–4000 nephelometric turbidity units, NTU) in red dye solutions (concentration from 0 to 2 mL diluted in water). Transmission detection of light in the: (**a**) 430 nm; (**b**) 522 nm; (**c**) 660 nm and (**d**) 870 nm wavelengths was performed.

In presence of turbid solutions, color determination with a direct calculation of absorbance cannot be performed. In colored turbid solutions, two different elements are contributing in the decrease of light along the path of propagation: while the suspended particles are scattering light, the liquid is absorbing it. In Figure 4 this can be seen clearly by experimental data, where both turbidity and the color concentration of the solution are increased. Here, red dye was used as an example of liquid absorber agent where volumes of 0.5, 1, 1.5 and 2 mL were added to 500 mL of water. For increased turbidity, corn starch was added to all the solutions to promote scattering centers. The size of these particles is on average 20 μm [17] where forward scattering is dominant [18]. Both chemical species were homogenous distributed in the solution. Measurements were carried with a constant temperature of 25 °C. As observed in Figure 4, while color attenuation is described by the Beer-Lambert law model, turbidity attenuation will present a similar behavior. Red dye was chosen as an example because it can be clearly seen the effect of light absorption in the 430 and 522 nm wavelengths while the 660 and 870 nm are barely affected.

Note that the turbidity measurement is in formazine nephelometric units (FNU), a measurement of relative clarity that follows the ISO 7072 standard [19]. FNU uses an infrared output, as opposed to the broadband light output used by turbidity meters that measure in nephelometric turbidity units (NTU). NTU and FNU will behave similarly when calibrated with formazine because of its common nephelometric technology.

The color absorption can be calculated separately of turbidity if the variation of light from the latter is already known and if the turbidity value is calculated by a way that is unaffected by the color. As can be seen in the case of Figure 4d, the infrared measurements are insensitive to changes in the dye concentration. This curve can be used to calculate turbidity separately from color. Knowing that attenuation of two different chemical species is the summation of the species attenuation separately, one can state that the total attenuation (*A_tot_*) is the sum of the turbidity attenuation (*A_tb_*) and color absorption (*A_col_*),

(4) $$A_{tot}=A_{tb}+A_{col}$$

From Equation (4) we can correlate it with the total light intensity detected (*I_tot_*) and with the expected light intensity obtained when in presence only with the turbidity (*I_tb_*). Therefore, when submerged in this two-chemical species solution *A_col_* can be calculated as:

(5) $$A_{tb}+A_{col}=-\log_{10}\left(\frac{I_{tot}}{I_{o}}\right)\Leftrightarrow A_{col}=\log_{10}\left(\frac{I_{tb}}{I_{o}}\right)-\log_{10}\left(\frac{I_{tot}}{I_{o}}\right)\Leftrightarrow A_{col}=-\log_{10}\left(\frac{I_{tot}}{I_{tb}}\right)$$

**Figure 5:**
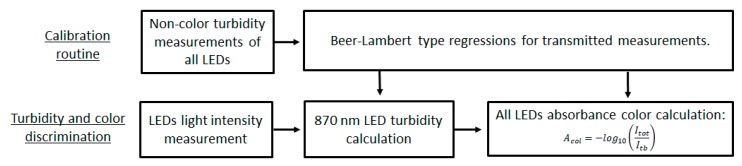
Schematic diagram for the process used to discriminate color from turbidity. Previous calibrations for turbidity are necessary.

This calculation was performed with the experimental data presented in Figure 4. To discriminate color from turbidity, a calibration routine of the sensor using all the wavelengths must be performed with variations of turbidity. The 870 nm wavelength is used to calculate the turbidity value since is unaffected by the color. Knowing the turbidity value using this wavelength and using the previous calibration data discussed, it is possible to calculate the expected light intensity for the other wavelengths with the same turbidity, but without the color absorption (*I_tb_*). Therefore, by calculating the real light intensity detected (*I_tot_*), this is, the one that is under attenuation and absorption of color and turbidity respectively, and using Equation (5), the color absorption can be easily calculated for each individual wavelength (Figure 5).

**Figure 6:**
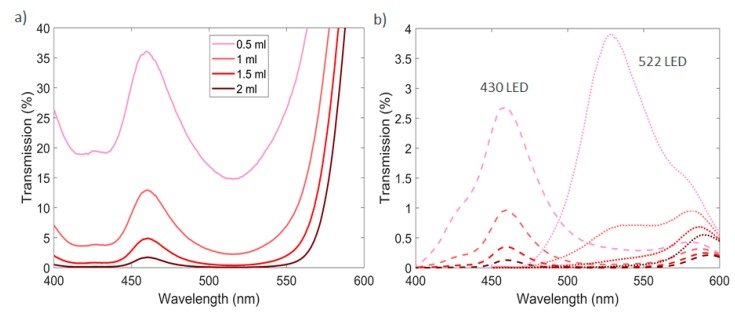
(**a**) Spectrographic measurements of red dye solution with different dye concentrations. (**b**) Real intensity detected by the sensor transmission photodetector based on its responsivity at the measured wavelengths.

A comparison of these experimental results with the theoretical expected absorption values obtained with the increasing of the red dye concentration in water solutions was performed based in spectrographic measurements taken and presented in Figure 6a. Considering the spectra given by 430 and 522 nm LEDs and the responsivity of the photodetector with wavelength, the real detected intensity expected by the sensor in its transmission photodetector was calculated and presented in Figure 6b.

By integrating the light intensity expected in the transmission photodetector with wavelength, the total expected normalized transmitted values can be obtained, and the absorbance deduced for each dye concentration. Using the methodology presented in Equation (5), a comparison between the experimental color absorbance and the expected theoretical ones obtained with turbid solutions is presented in Table 1.

The values for the absorbance obtained by the chromatic sensor are close to what was expected theoretically from the deduced values from the spectrophotometer. But the real comparison must be performed with the values obtained for 0 FNU, i.e., without turbidity. Accuracies of about ±0.3 absorbance units (AU) are obtained in turbid liquids. It is important to notice that for low color concentrations, but high turbidity values (turbidity dominance), the error deviation of the calculated color is higher than when high color concentration is present (color dominance). In this region of measurement, color absorbance presents very stable calculated values. Overall, the turbidity compensation for color measurement is achieved.

## 4. Turbidity and Refractive Index

**Figure 7:**
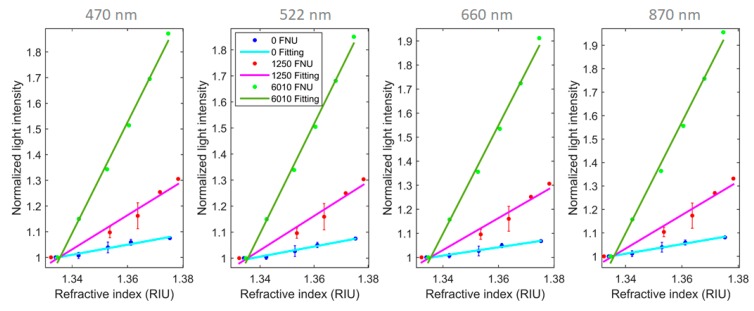
Experimental light intensity variation obtained with the increase of the refractive index units (RIU) and normalized to the refractive index of water for solutions with 0, 1250 and 6010 FNU for all the sensor transmission light sources channels. Linear regressions to the experimental data is also presented with its parameters and root-mean-square deviation (RMSE) calculated in Table 2.

Refractive index (RI) changes, in clear or turbid solutions, will create variations in the transmitted channel. Contrary to color and turbidity that have a negative contribution for the light detected with increase of absorber and scattering centers, a refractive index increase will have a positive contribution in light-intensity detection. Depending on the concentration of particles present in the solution, it will have different increasing rates. In Figure 7, a representation of this variation measured with the sensor transmission channel at 25 °C is visible for solutions prepared with turbidities of 0, 1250 and 6010 FNU using corn starch. The refractive index was changed by adding sucrose to the prepared solutions and measured with a Abbemat 200 refractometer from Anton Paar.

**Figure 8:**
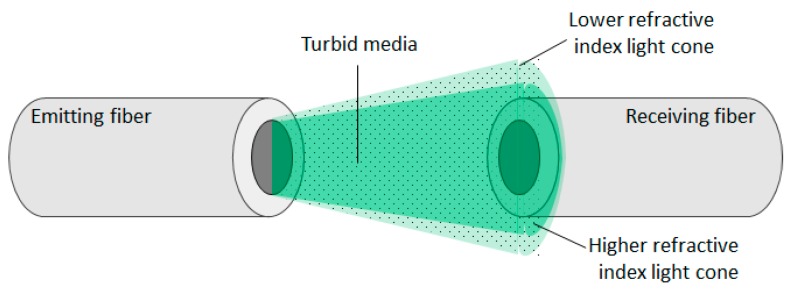
Scheme of the created light cone from an emitting fiber in turbid media with lower and higher refractive indices. The difference of light variation detected in the receiving fiber will be dependent on the concentration of particles.

The different increasing variation rates in the detected light observed, in contrast to that obtained from clear solutions, is explained considering the light cone created in the exit of the emitting fiber. It will decrease its aperture with the increase of the refractive index of the solution (*n_ext_*) as seen in Equation (2), and consequently focus more light into the receiving fiber, increasing its intensity value. Adding to this effect, the low aperture cone will also interact with less scattering centers and therefore, effectively, it will detect less turbidity than with a lower RI solution (Figure 8).

**Figure 9:**
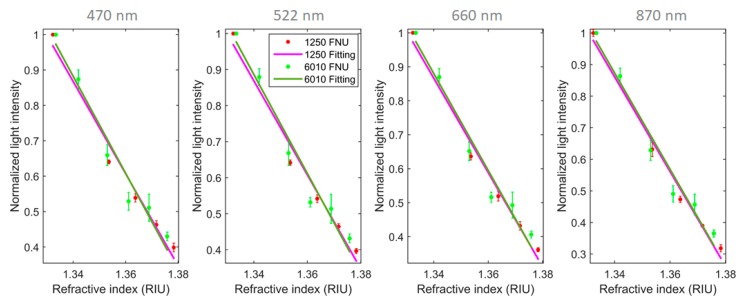
Experimental light intensity variation obtained with the increase of the refractive index and normalized to the refractive index of water for solutions with 1250 and 6010 FNU for all the sensor scattering light sources channels.

In the receiver fiber dedicated to the scattered light, there is no effect in intensity when measuring variations of refractive index in clear solutions, due to light not achieving this fiber. In turbid media the light will reach the scattering channel and, therefore, changes in the geometry of the emitting light cone will have effects in the detected scattering light. As discussed, with a more focused light in the transmission channel, less light will be scattered and detected in the related channel. Again, adding to this effect, with a lower angle light cone there are fewer interactions with the particles to scatter light and so, as a result, even less light is scattered. This will be expressed as a decreasing variation in the detected light with the increase of the refractive index. Experimental values are presented in Figure 9 for all scattering channels with turbidities of 1250 and 6010 FNU.

**Figure 10:**
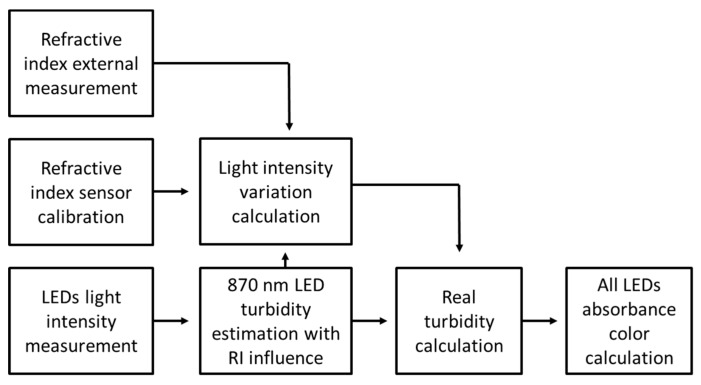
Schematic diagram for possible process to be used for refractive index compensation.

To compensate this phenomenon for posterior calculation of color and turbidity, external measurement of refractive index value using another sensor will need to be performed, but effective compensation for this sensor can be achieved using interpolation values from Table 2. The process starts with the calculation of turbidity using the 870 nm LED as described in Figure 10. Knowing this value of turbidity, the value or refractive index and executing an interpolation from the Table 2 values, the variation of light intensity signal from this solution in relation to a solution with refractive index of water can be assessed. The variation assessment will lead to the real turbidity value of the solution and the latter to the real color absorption value (Figure 10).

## 5. Conclusions

An in-line and real time chromatic sensor was developed using the air gap fiber-to-fiber transmission principle. The experimental data obtained with the variation of color and turbidity has shown that a differentiation of the two elements can be obtained if the behavior of the turbidity without color is known. It is achieved by having an independent measurement of turbidity without color influence using infrared light. A compensation routine is performed and an accuracy of about ±0.3 AU is attained. It was also shown that linear variations of light will be observable with the increase of the refractive index for both clear and turbid solutions. To compensate for this parameter, discussion is required of how to linearly adjust this variation after the first turbid estimation where an independent refractive index-sensing mechanism is needed.

The study here presented could lead to the development of more robust data fusion and machine-learning algorithms that have the potential to use the multivariate data to combine and analyze the information in order to provide more accurate measurement.

## Figures and Tables

**Table 1:** Comparison between the theoretical expected color absorbance and the detected absorbance of a red dye solutions with variations in turbidity in formazine nephelometric units (FNU).

Dye Concentration (mL)		430 nm LED				522 nm LED			
		0.5	1.0	1.5	2.0	0.5	1.0	1.5	2.0
**Theoretical Absorbance**		0.61	1.13	1.55	1.86	0.63	1.32	1.72	1.90
Sensor Absorbance	0 FNU	0.58	1.10	1.62	2.12	0.66	1.24	1.77	2.21
	164 FNU	0.60	1.08	1.65	2.12	0.69	1.22	1.81	2.22
	449 FNU	0.55	1.11	1.66	2.12	0.64	1.26	1.83	2.22
	999 FNU	0.50	1.03	1.57	1.96	0.60	1.19	1.75	2.06
	2020 FNU	0.46	0.99	1.61	2.06	0.52	0.95	1.80	2.20
	3571 FNU	0.38	0.82	1.67	2.20	0.36	0.85	1.89	2.41

**Table 2:** Linear regression parameters obtained from the experimental values observed in Figure 7.

Turbidity Value (FNU)		Linear Model: y = ax + b		R^2^	RMSE
		a	b		
470 nm	0	1.962	−1.618	0.9668	0.0068
	1250	6.649	−7.879	0.9625	0.0273
	6010	17.29	−22.07	0.9952	0.0217
522 nm	0	1.950	−1.607	0.9741	0.0059
	1250	6.575	−7.780	0.9613	0.0274
	6010	17.01	−21.71	0.9957	0.0201
660 nm	0	1.741	−1.325	0.9799	0.0046
	1250	6.653	−7.885	0.9600	0.0282
	6010	18.10	−23.16	0.9956	0.0218
870 nm	0	2.052	−1.739	0.9894	0.0040
	1250	7.160	−8.562	0.9608	0.0300
	6010	19.42	−24.93	0.9941	0.0270
